# Association between Early Acute Respiratory Distress Syndrome after Living-Donor Liver Transplantation and Perioperative Serum Biomarkers: The Role of Club Cell Protein 16

**DOI:** 10.1155/2019/8958069

**Published:** 2019-04-11

**Authors:** Chun-Yu Wu, Ya-Jung Cheng, Ming-Hui Hung, I-Ju Lin, Wei-Zen Sun, Kuang-Cheng Chan

**Affiliations:** Department of Anesthesiology, National Taiwan University, College of Medicine and Hospital, Taipei, Taiwan

## Abstract

**Background:**

Acute respiratory distress syndrome (ARDS) after living-donor liver transplantation (LDLT) is not uncommon, but it lacks the biomarkers for early detection. Club cell protein 16 (CC16), high-motility group box 1 protein (HMGB1), interleukin-1*β* (IL-1*β*), and IL-10 have been reported as relevant to the development of ARDS. However, they have not been investigated during LDLT.

**Methods:**

Seventy-three consecutive recipients undergoing LDLT were enrolled and received the same perioperative care plan. Perioperative serum CC16, HMGB1, IL-1*β*, and IL-10 levels were measured at the pretransplant state, 30 minutes after reperfusion, postoperative day 1 (POD1), and POD3. ARDS was diagnosed according to the 2012 Berlin definition.

**Results:**

Of the 73 recipients, 13 developed ARDS with significantly longer durations of mechanical ventilation and intensive care unit stay. Serum CC16 levels on POD1 increased significantly from the pretransplant state in the ARDS group but not in the non-ARDS group. Pretransplant serum CC16 levels were also higher in the ARDS group. The area under the receiver operating characteristic curves for POD1 serum CC16 levels used to discriminate ARDS was 0.803 (95% confidence interval: 0.679 to 0.895; p < 0.001). By comparison, HMGB1, IL-1*β*, and IL-10 were not associated with ARDS after LDLT.

**Conclusion:**

The higher pretransplant serum CC16 level and its increased level on POD1 were associated with the development of early ARDS after LDLT. This trial is registered with NCT01936545, 27 August 2013.

## 1. Introduction

Early acute respiratory distress syndrome (ARDS) in the first 72 hours after living-donor liver transplantation (LDLT) is not uncommon [1, 2] because liver transplantation (LT) is associated with ischemia reperfusion–induced profound inflammatory responses [3–6]. Studies have mainly focused on pretransplant factors such age [1, 7] and high Model for End-Stage Liver Disease (MELD) scores [6] for predicting the development of ARDS after LDLT. By contrast, there is a paucity of research investigating the serum biomarkers of lung inflammation for early detection of ARDS after LDLT.

Club cell protein 16 (CC16) is a lung secretory protein [8] with antioxidant and anti-inflammatory properties. During lung inflammation, lung secretory proteins move passively across the epithelial barrier into serum [8]; accordingly, increases of CC16 in serum have been reported in both animal models of [9] and human studies [10–12] on ARDS. Moreover, serum CC16 has been reported as a sensitive biomarker that may discriminate lung injury in surgical patients within hours [13, 14]. Therefore, monitoring perioperative serum CC16 levels may be valuable for early detection of ARDS after LDLT. However, this remains uninvestigated.

Furthermore, several potent proinflammatory cytokines such high-mobility group box 1 protein (HMGB1) [15] and interleukin-1*β* (IL-1*β*) [15, 16] and potent anti-inflammatory cytokines such as IL-10 [17–19] are known to participate in the development of or protection from lung injury. However, the association between these biomarkers and early ARDS after orthotropic LT has not been investigated.

In this prospective observational study, our aim was to clarify the role of potential serum biomarkers, namely, CC16, HMGB1, IL-1*β*, and IL-10, in the development of ARDS after LDLT.

## 2. Methods

### 2.1. Patients

After receiving Institutional Review Board approval (NCT201003116M) and registration at “ClinicalTrial.gov” (NCT01936545) for our study protocol and written informed consent from patients at National Taiwan University Hospital, we consecutively enrolled 73 adult patients with end-stage liver disease who were scheduled to receive LDLT. The exclusion criteria were as follows: age younger than 20 years, a history of pulmonary resection, chronic respiratory insufficiency, cardiac dysfunction (as determined by preoperative echocardiography), and surgery failure.

### 2.2. Perioperative Management

We followed the methods of Chan et al. 2017 [20]. General anesthesia was regulated by maintaining the bispectral index between 40 and 60 during surgery. Intraoperative mechanical ventilation was set at a tidal volume of 8 mL kg^–1^ (based on ideal body weight) and a respiratory rate of 10–20 min^–1^ to maintain normocapnia and a positive end-expiratory pressure (PEEP) of 5 cmH_2_O. The maximal peak inspiratory pressure was set at 35 cmH_2_O. Perioperative hemodynamic status was monitored and maintained using a pulse index contour continuous cardiac output monitor (Version 7.0; Pulsion Medical Systems, Feldkirchen Germany) [21]. The LDLT procedure was performed using the piggyback technique without venovenous bypass [2]. Decisions regarding the administering of fluids and blood products were made according to the same standards of care, to provide hemodynamic stability and to correct unexpected coagulation abnormalities and bleeding. Temporary dopamine infusion or norepinephrine boluses were administered to maintain the mean arterial pressure above 65 mmHg intraoperatively.

During the perioperative period, the patients were kept on an immunosuppression regimen, namely, tacrolimus, prednisolone, and basiliximab, based on the LDLT protocol in our hospital. The trough level of tacrolimus was maintained between 7 and 10 ng/mL for the first month after LDLT and between 5 and 7 ng/mL thereafter. Steroids were gradually tapered within the first month after LDLT. An IL-2 receptor blocker, such as basiliximab (Simulect), was infused on the day of LDLT surgery and on day 4 after surgery.

All patients were admitted to the same intensive care unit (ICU) and received the same respiratory care and weaning protocol of mechanical ventilation. The initial mechanical ventilator settings were FiO_2_ 60%, a tidal volume of 8 mL kg^–1^ (based on ideal body weight), a respiratory rate of 12–20 min^–1^, and a PEEP of 5 cmH_2_O. The maximal peak inspiratory pressure was set at 35 cmH_2_O. The FiO_2_ and PEEP were titrated according to regular arterial blood gas analysis every 8 h. Chest radiography was performed daily during the ICU stay. Patients were extubated in the ICU according to the same weaning criteria, namely, clear consciousness, a clean chest radiograph, rapid shallow breathing index, and respiratory rate/tidal volume ratio ≤ 105 breaths/min/L (tidal volume 5 mL kg^−1^, frequency less than 30 breaths/min, and maximal inspiratory pressure or negative inspiratory force less than 30 cmH_2_O). During the ICU stay, echocardiography was conducted to eliminate cardiogenic pulmonary edema in patients with suspected clinical signs and symptoms.

### 2.3. Definition of Acute Respiratory Distress Syndrome after Liver Transplantation

Postoperative ARDS was diagnosed according to the 2012 Berlin definition of ARDS, specifically PaO_2_/FiO_2_ < 300 and the acute onset of bilateral infiltrates on the chest radiograph during postoperative day 1 (POD1) to POD3, which were not fully explained by cardiac failure [22–24]. ARDS was based on degree of hypoxemia: mild (200 mm Hg < PaO2/FIO2 ≤ 300 mm Hg), moderate (100 mm Hg < PaO2/FIO2 ≤ 200 mm Hg), and severe (PaO2/FIO2 ≤ 100 mm Hg). Chest radiographs were evaluated by two independent radiologists according to the same scoring system.

### 2.4. Measurement of Serum Biomarkers

Plasma levels of the biomarkers, namely, CC16, HMGB1, IL-1*β*, and IL-10, were measured and compared between the baseline (T_1_, after anesthesia induction), 30 min after reperfusion (T_2_), POD1 (T_3_), and POD3 (T_4_). Serum concentrations of HMGB1 (Chondrex Inc., Redmond, WA, USA), CC16 (BioVendor LLC, Candler, NC, USA), IL-1*β* (BioLegend, San Diego, CA, USA), and IL-10 (BioLegend, San Diego, CA, USA) were measured using enzyme-linked immunosorbent assay kits.

### 2.5. Statistical Analysis

Data analyses were performed by statisticians who were blinded to the purpose of this study. The sample size was estimated to detect serum CC16 levels with a difference of 7.5 ng/mL [10] with an incidence of ARDS after LDLT of 20% [1] because among these measured biomarkers, CC16 is the most commonly reported in relation to ARDS. Accordingly, nine patients with ARDS and 36 patients without ARDS were determined from the estimation. Analysis of variance (ANOVA) of repeated measurements was performed to assess the significance of differences in means between and within the groups, and post hoc analysis using the Tukey method was performed if any time effects or time-group interactions were significant [25]. The Youden index was maximized in the area under the receiver operating characteristic curves (AUROCs) to calculate potential variables that discriminate ARDS at the pretransplant status [26]. Hypothesis testing was two-tailed, with a significance level of p < 0.05 and statistical power of >0.8. The MedCalc program (MedCalc Inc., Mariakerke, Belgium) was used to perform all statistical analyses and to plot graphs.

## 3. Results

### 3.1. Pretransplant Characteristics of Patients

Table 1 presents a summary of the patient characteristics. The most common etiology of end-stage liver disease was viral hepatitis. Among the 73 patients, 13 developed ARDS after LDLT (17.8%), consisting of 8 patients with mild ARDS, 4 patients with moderate ARDS, and 1 patient with severe ARDS. Patients in the ARDS group were older (58.8 ± 6.1 vs. 52.6 ± 9.6 y; p = 0.0287) than patients in the non-ARDS group; both the ARDS and non-ARDS groups of recipients had comparable sex ratios and pretransplant serum albumin levels, MELD scores, lung function test results, and intrathoracic blood volume indices. Echocardiography revealed no pretransplant cardiac dysfunction, such as left ventricle failure, in any patient.

### 3.2. Intraoperative Profiles and Postoperative Outcomes

The ARDS and non-ARDS groups were comparable in terms of operation time (558.1 ± 95.6 vs. 537.6 ± 91.3 min; p = 0.4689), anhepatic phase duration (67.6 ± 19.1 vs. 62.9 ± 20.6 min; p = 0.4572), intraoperative blood loss (3765.4 ± 3450.1 vs. 2535.8 ± 2827.5 mL; p = 0.1762), intraoperative intravenous fluid volume (3850.0 ± 3230.6 vs. 3064.8 ± 1909.7 mL; p = 0.4125), amount of albumin administered, and amount of blood products transfused (Table 2).

Compared with recipients in the non-ARDS group, those in the ARDS group had a prolonged mechanical ventilation duration (6.7 ± 8.0 vs. 1.2 ± 0.5 d, p = 0.0291) and ICU stay (13.9 ± 9.8 vs. 6.9 ± 1.2 d, p = 0.0238) and a comparable hospital stay (37.4 ± 21.7 vs. 28.9 ± 13.2 d, p = 0.1954).

### 3.3. Serum Biomarkers Levels in Patients with and without Acute Respiratory Distress Syndrome

**Figure 1:**
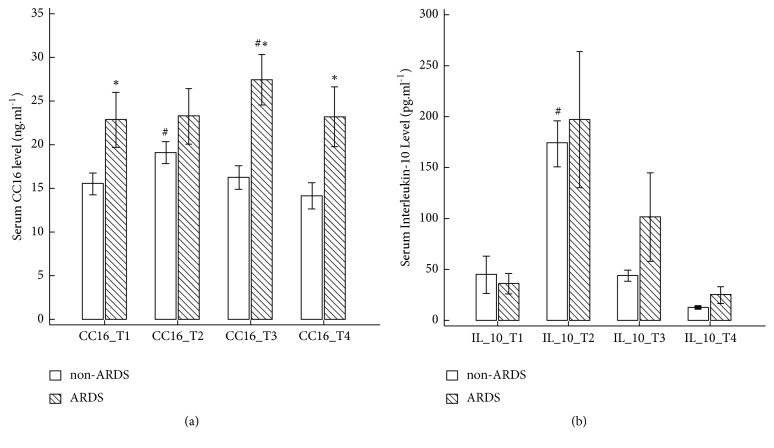
Changes in perioperative serum anti-inflammatory biomarkers consisting of club cell protein 16 (CC16) (Figure 1(a)) and interleukin-10 (IL-10) (Figure 1(b)) in nonacute respiratory distress syndrome (ARDS) and ARDS groups. *∗* indicates a significant difference of p < 0.05 between the groups. # indicates a significant change compared with the pretransplant state of p < 0.05 within the groups.

ANOVA results showed that serum CC16 levels were significantly different between patients in the ARDS group and those in the non-ARDS group (group effect p = 0.003, time effect p = 0.022, group-time interaction p = 0.026; Figure 1(a)). Serum CC16 levels were significantly higher in the ARDS group than in the non-ARDS group in pretransplant and postoperative measured time points (T_1_ [the pretransplant state]: 22.9 ± 10.4 vs. 15.5 ± 8.7 ng.mL^−1^; T_2_ [POD1]: 27.5 ± 9.6 vs. 16.3 ± 9.4 ng.mL^−1^; T_3_ [POD3]: 23.2 ± 11.4 vs. 14.2 ± 10.5 ng.mL^−1^; T_4_ [POD3]: 14.2 ± 10.5 vs. 23.2 ± 11.4 ng.mL^−1^; all p < 0.05; Figure 1(a)). Hepatic reperfusion caused a significant increase in serum CC16 in the non-ARDS group but not in the ARDS group. By contrast, serum CC16 significantly increased in the ARDS group but not in the non-ARDS group on POD1 (Figure 1(a)).

The AUROCs of serum CC16 levels on POD1 were 0.803 (95% confidence interval: 0.679 to 0.895; p < 0.001; Figure 3). The cutoff value for serum CC16 levels on POD1 with the highest Youden index was 16.8 ng/mL (sensitivity: 91%, specificity: 60%) and 27.3 ng/mL (sensitivity: 55%, specificity: 96%).

**Figure 2:**
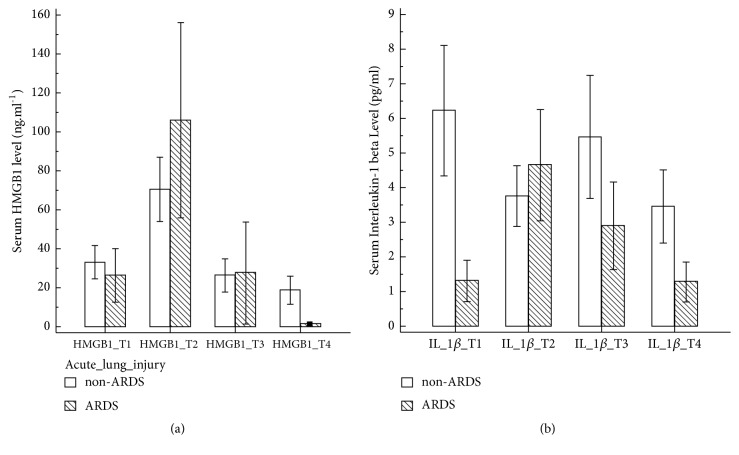
Changes in perioperative proinflammatory biomarkers consisting of high-motility group box 1 protein (HMGB1) (Figure 2(a)) and interleukin-1*β* (IL-1*β*) (Figure 2(b)) in nonacute respiratory distress syndrome (ARDS) and ARDS groups.

**Figure 3:**
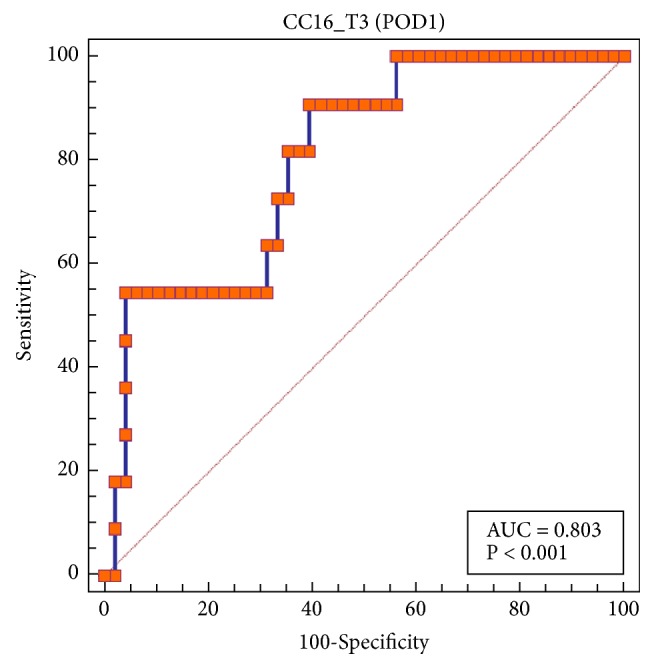
Receiver operating characteristic curves describing the ability of serum club cell protein 16 (CC16) levels early in the morning on postoperative day 1 in discriminating early postoperative acute respiratory distress syndrome.

After hepatic reperfusion (T_2_), the non-ARDS group had a significant increase in IL-10 (173.2 ± 155.8 vs. 44.3 ± 126.6 pg.mL^−1^ at T_2_ and T_1_, respectively; p = 0.0002; Figure 1(b)) and a trend of increased HMGB1 (70.5 ± 112.8 vs. 33.2 ± 58.8 ng.mL^−1^ at T_2_ and T_1_, respectively; p = 0.0884; Figure 2(a)) compared with the baseline (T_1_) values. By contrast, there was no significant serum IL-1*β* change in both the ARDS and non-ARDS groups (Figure 2(b)). Furthermore, no associations between early ARDS after LDLT and other biomarkers, namely, HMGB1, IL-1*β*, and IL-10, were observed.

## 4. Discussion

The main finding of this study is that ARDS after LDLT was associated with higher preoperative and postoperative serum CC16 levels. By contrast, the perioperative serum HMGB1, IL-1*β*, and IL-10 levels were not associated with early ARDS after LDLT.

We found that only patients in the ARDS group had increased serum CC16 levels early in the morning of POD1. Because LDLT is associated with a significant amount of intraoperative bleeding and transfusion, particularly when red blood cells and plasma are transfused, it leads to liver ischemia-reperfusion injury, which increases the levels of multiple inflammatory mediators that become active in the lungs [6, 27, 28]. Subsequently, inflammatory interactions between the liver and lungs as well as the activation of nuclear factor *κ*B [29, 30] may further contribute to the pathogenesis of permeability-type pulmonary edema, one of the most common etiologies of early ARDS after LDLT [2]. Because CC16 acts to protect the integrity of the epithelial lining against inflammation and oxidant stress [8], the elevation of serum CC16 was detectable during injury, which may indicate a protective response to intraoperative ischemia-reperfusion insults. In this study, the serum CC16 level (approximately 20 ng.mL^−1^) in recipients with ARDS is similar to those previously reported in ARDS patients [31]. In ARDS patients, Lesur et al. reported that serum CC16 levels were higher in nonsurvivors from days 2–14, and higher CC16 levels were associated with fewer days free of the ventilator, as well as the increased frequency and severity of nonpulmonary organ failure [32]. Our results are congruent with those reported by Lesur et al., whereby higher serum CC16 levels were noted in patients in ARDS groups who had worse outcomes such as prolonged mechanical ventilator support and longer ICU stays compared with other patients. In addition, we found that a lower serum CC16 level (16.8 nL/mL) is associated with a high sensitivity (91 %) and a higher serum CC16 level (27.3 ng/mL) is associated with high specificity (96 %) to detect ADRS; this biomarker may provide useful information for clinician to include or exclude early ARDA after LDLT.

In addition to the postoperative increase in serum CC16, we found that pretransplant serum CC16 levels were higher in the ARDS group than in the non-ARDS group. Shah et al. suggested that pretransplant CC16 levels are associated with preexisting epithelial injury before LT, which increases the likelihood of postoperative graft failure [33]. Our results agree with this finding, because patients in our ARDS group also presented with a significantly higher pretransplant serum CC16 level. In addition, age has been identified as a risk factor for postoperative pulmonary complications and poor survival rates [1, 34] because older patients are at an increased risk of comorbid conditions. Therefore, higher pretransplant lung epithelial damage presenting with a higher serum CC16 level is found among older patients.

A transient increase in IL-10 after hepatic reperfusion was observed in our patients, which is compatible with the finding of another study [35] that reported that accumulating macrophages after reperfusion secrete IL-10 [36, 37]. Because macrophages can be largely affected by the immunosuppressive drugs for preventing allograft rejection and because macrophages have divergent roles in solid organ transplantation, including both harmful and protective effects [38], the association between IL-10 and early ARDS after LDLT may be diluted.

Although studies have reported that HMGB1 is a pertinent biomarker in the development of lung injury in experimental models [16, 39] as well as a potentially important biomarker in ARDS [15], our study did not observe this association. Because potent immunosuppressant drugs were required for transplantation surgery, the role of perioperative proinflammatory biomarkers for detecting or predicting ARDS may have been diluted. Therefore, neither HMGB1 nor IL-1*β* was associated with the early ARDS after LDLT in our patients. However, an increasing trend of HMGB1 after hepatic reperfusion was observed, which was not identified in IL-1*β*. This is compatible with the findings of previous studies that have reported that HMGB1 is associated with liver ischemia-reperfusion injury in an experimental model [40] and is a useful biomarker of hepatocellular injury in LT [41]. This result suggests that HMGB1 may play a role in the detection of acute hepatic reperfusion injury.

Although this is the first study to report the possible role of serum CC16 levels in ARDS after LDLT, it is limited by its modest sample size. Large-scale validation is necessary to identify more potential variables.

In conclusion, our study showed that serum CC16 levels increased early in the morning of POD1 in recipients who developed ARDS but not in those without ARDS. Moreover, higher pretransplant serum CC16 levels were noted in recipients who developed ARDS, which correlated with the pretransplant severity of liver cirrhosis. Therefore, monitoring serum CC16 may provide information for predicting early ARDS after LDLT.

## Figures and Tables

**Table 1:** Pretransplant patient characteristics.

	with ARDS (N=13)	without ARDS (N=60)	P value
Age (yr)	58.8 ± 6.1	52.6 ± 9.6	0.0287

Gender (Male, %)	6(46.15%)	38(63.33%)	0.2511

BMI (kg/m^2^)	25.8 ± 4.5	24.0 ± 3.9	0.1854

Serum albumin level (g/dL)	3.0 ± 0.6	3.3 ± 0.8	0.1227

Etiologies of end-stage liver disease			
Viral hepatitis	9(69.2%)	44(73.3%)	0.7652
Others	4(30.8%)	16(26.7%)	

MELD score	18 ±10.2	14.8 ± 8.1	0.2158

FVC, % of prediction	86.8 ± 24.7	90.3 ± 18.3	0.5782

FEV_1_, % of prediction	88.0 ± 25.8	90.0 ± 19.0	0.7614

BMI = body mass index; ARDS = acute respiratory distress syndrome; MELD = model for end-stage liver disease; FVC = forced vital capacity; FEV_1_ = forced expiratory volume in 1 second.

**Table 2:** Intraoperative profiles and the intensive care stay.

	with ARDS (N=13)	without ARDS (N=60)	T-test P value
Operation time (min)	558.1 ± 95.6	537.6 ± 91.3	0.4689

Anhepatic time (min)	67.6 ± 19.1	62.9 ± 20.6	0.4572

Blood loss (ml)	3765.4 ± 3450.1	2535.8 ± 2827.5	0.1762

Transfusion			
PRBC (U)	14.7 ± 12.0	7.5 ± 7.9	0.0571
FFP (U)	7.6 ± 6.8	5.2 ± 7.5	0.2933
Platelet (U)	21.2 ± 18.4	16.6 ± 17.9	0.407

Albumin usage (bot)	4.62±0.65	4.7±0.72	0.6976

Intravenous fluid (ml)	3850.0 ± 3230.6	3064.8 ± 1909.7	0.4125

Mechanical ventilation duration (d)	6.7 ± 8.0	1.2 ± 0.5	0.0291

ICU stay (d)	13.9 ± 9.8	6.9 ± 1.2	0.0238

Hospital stay (d)	37.4 ± 21.7	28.9 ± 13.2	0.1954

Anesthetic			0.1131
Desflurane	9(69.23%)	27(45%)	
Propofol	4(30.77%)	33(55%)	

ARDS = acute respiratory distress syndrome; PRBC = packed red blood cell; FFP = fresh frozen plasma; MV = mechanical ventilation; ICU = intensive care unit.

## Data Availability

All data created during this research are available from the corresponding author upon request.
